# Patterns of homozygosity in insular and continental goat breeds

**DOI:** 10.1186/s12711-018-0425-7

**Published:** 2018-11-19

**Authors:** Taina F. Cardoso, Marcel Amills, Francesca Bertolini, Max Rothschild, Gabriele Marras, Geert Boink, Jordi Jordana, Juan Capote, Sean Carolan, Jón H. Hallsson, Juha Kantanen, Agueda Pons, Johannes A. Lenstra, The AdaptMap Consortium

**Affiliations:** 1 Department of Animal Genetics, Centre for Research in Agricultural Genomics (CRAG), CSIC-IRTA-UAB-UB, Campus de la Universitat Autònoma de Barcelona, 08193 Bellaterra, Barcelona Spain; 2 CAPES Foundation, Ministry of Education of Brazil, Brasília, DF 70.040-020 Brazil; 3 Departament de Ciència Animal i dels Aliments, Facultat de Veterinària, Universitat Autònoma de Barcelona, 08193 Bellaterra, Spain; 4 Department of Animal Science, Iowa State University, Ames, IA 50011-3150 USA; 5 Bioinformatics Core Facility, Fondazione Parco Tecnologico Padano, Loc. Cascina Codazza, 26900 Lodi, LO Italy; 6 Stichting Zeldzame Huisdierrassen, De Drieslag 30, 8251 JZ Dronten, The Netherlands; 7 Instituto Canario de Investigaciones Agrarias, 38108 La Laguna, Tenerife Spain; 8 The Old Irish Goat Society, Mulranny, Co Mayo Ireland; 9 Faculty of Land and Animal Resources, Agricultural University of Iceland, Reykjavík, Iceland; 10 Department of Production Systems, Natural Resources Institute Finland, 31600 Jokioinen, Finland; 11 Unitat de Races Autòctones, Servei de Millora Agrària i Pesquera (SEMILLA), 07198 Son Ferriol, Spain; 12 Faculty of Veterinary Medicine, Utrecht University, Utrecht, The Netherlands

## Abstract

**Background:**

Genetic isolation of breeds may result in a significant loss of diversity and have consequences on health and performance. In this study, we examined the effect of geographic isolation on caprine genetic diversity patterns by genotyping 480 individuals from 25 European and African breeds with the Goat SNP50 BeadChip and comparing patterns of homozygosity of insular and nearby continental breeds.

**Results:**

Among the breeds analysed, number and total length of ROH varied considerably and depending on breeds, ROH could cover a substantial fraction of the genome (up to 1.6 Gb in Icelandic goats). When compared with their continental counterparts, goats from Iceland, Madagascar, La Palma and Ireland (Bilberry and Arran) displayed a significant increase in ROH coverage, ROH number and *F*_ROH_ values (*P* value < 0.05). Goats from Mediterranean islands represent a more complex case because certain populations displayed a significantly increased level of homozygosity (e.g. Girgentana) and others did not (e.g. Corse and Sarda). Correlations of number and total length of ROH for insular goat populations with the distance between islands and the nearest continental locations revealed an effect of extremely long distances on the patterns of homozygosity.

**Conclusions:**

These results indicate that the effects of insularization on the patterns of homozygosity are variable. Goats raised in Madagascar, Iceland, Ireland (Bilberry and Arran) and La Palma, show high levels of homozygosity, whereas those bred in Mediterranean islands display patterns of homozygosity that are similar to those found in continental populations. These results indicate that the diversity of insular goat populations is modulated by multiple factors such as geographic distribution, population size, demographic history, trading and breed management.

**Electronic supplementary material:**

The online version of this article (10.1186/s12711-018-0425-7) contains supplementary material, which is available to authorized users.

## Background

The advent of next-generation sequencing and high throughput genotyping techniques has made it possible to identify, in the genomes of multiple species, continuous homozygous stretches of sequence, which are named runs of homozygosity (ROH) [1]. The genomic distribution, abundance, and length of ROH are modulated by multiple factors including local recombination rate, guanine-cytosine content, positive selection and demography [2, 3]. A high frequency of long ROH is often caused by recent inbreeding, whereas a high frequency of short ROH can be explained by the occurrence of an ancient founder effect or population bottleneck. After the first pioneering study of Ferenčaković et al. [4], the patterns of ROH have been characterized for multiple domestic species and breeds with the goal of making inferences about their history and demography as well as of identifying the genomic footprint of natural and artificial selection [5].

Geographic isolation of populations may lead to a considerable loss of diversity, an increase in inbreeding and vulnerability to stochastic events [6]. For instance, human populations with a history of prolonged isolation on the Orkney or Dalmatian Islands or in Sardinia have longer ROH than continental populations, which indicates an elevated relatedness [7, 8]. A high frequency of ROH can have detrimental effects on biological fitness and reproductive success because ROH are often enriched in deleterious mutations [9]. Indeed, mitochondrial encephalomyopathy is relatively frequent in people from the Faroe Islands due to homozygosity of the *SUCLA2* gene [10]. In cattle, Zhang et al. [11] reported that deleterious variations are overrepresented in ROH regions, particularly in those longer than 3 Mbp. However, geographically isolated populations may have retained ancient alleles or variants that are not found in other populations [12, 13], which reflects adaptation to harsh environments and/or a practice of breed management that are not common for mainland populations [13–15].

Recently, genome-wide single nucleotide polymorphism (SNP) data for a comprehensive panel of goats breeds has become available [16]. For the same panel of breeds, signatures of selection [17] and the effects of population size, breed management and crossbreeding on the patterns of ROH as well as chromosomal ROH hotspots have been reported [18]. The aim of our study was to investigate if goat breeds that are raised in islands have higher levels of homozygosity than their continental counterparts. In order to achieve this goal, we compared the number and genomic coverage of ROH in 25 caprine breeds from 16 European and African islands with those of nine continental populations.

## Methods

### Goat sampling and genotyping

Goats were sampled and genotyped as part of the AdaptMap project [19] and see Table 1. The geographic distribution of the breeds investigated in this study is illustrated in Figure S1 (see Additional file 1: Figure S1). Genomic DNA was extracted with standard protocols and goats were genotyped with the Illumina Goat SNP50 BeadChip [20] by following the manufacturer’s instructions. Monomorphic and unmapped SNPs in the whole dataset, and SNPs with a call rate lower than 98% were eliminated. Individuals with a genotype call rate lower than 96% were removed. Quality control was performed by using the PLINK program [21]. The final dataset included 46,654 SNPs and 480 goats with 260 and 220 individuals from insular and continental populations, respectively (Table 1) and (see Additional file 1: Figure S1). In order to calculate allele-sharing distances (ASD), linkage disequilibrium-pruning of SNPs was implemented (plink option—indep-pairwise 50 5 0.03) [21], which retained 19,879 SNPs.

**Table 1:** ROH number, ROH length and *F*_ROH_ mean, minimum (min) and maximum (max) values calculated for the 25 goat populations used in this study (SE = standard error)

Breed code	Breed name	Country	Number of animals	ROH length		ROH number		*F*_ROH_	
				Mean ± SE	Min/max	Mean ± SE	Min/max	Mean ± SE	Min/max
AND	Androy	Madagascar	6	478.44 ± 19.43	423.28/559.05	259.67 ± 7.88	235/289	0.19 ± 0.01	0.17/0.23
ARG	Argentata	Italy (Sicily)	24	47.01 ± 9.21	16.78/248.58	21.79 ± 1.15	13/35	0.02 ± 0.00	0.01/0.10
ARR	Arran	Ireland	8	733.63 ± 47.82	513.01/938.65	178.00 ± 7.05	147/209	0.30 ± 0.02	0.21/0.38
ASP	Aspromontana	Italy	23	135.05 ± 30.77	20.77/599.81	35.61 ± 4.40	15/102	0.05 ± 0.01	0.01/0.24
BLB	Bilberry	Ireland	10	533.28 ± 44.00	88.00/780.21	67.00 ± 5.33	23/114	0.22 ± 0.02	0.04/0.32
CCG	Ciociara Grigia	Italy	16	155.39 ± 29.90	7.83/362.79	30.63 ± 4.12	7/60	0.06 ± 0.01	0.00/0.15
CRS	Corse	France (Corsica)	29	93.72 ± 14.29	23.55/295.13	34.86 ± 1.68	18/65	0.04 ± 0.01	0.01/0.12
DIA	Diana	Madagascar	14	636.87 ± 60.53	481.75/1279.29	265.79 ± 4.88	229/292	0.26 ± 0.02	0.20/0.52
DKL	Danish Landrace	Denmark	50	401.18 ± 28.00	490.30/714.81	67.06 ± 1.94	111/141	0.16 ± 0.01	0.20/0.29
DUL	Dutch Landrace	Netherlands	15	622.60 ± 15.80	133.11/889.28	125.27 ± 1.96	41/102	0.25 ± 0.01	0.05/0.36
FIN	Finnish Landrace	Finland	20	225.46 ± 32.00	118.47/686.10	80.75 ± 2.77	65/122	0.09 ± 0.01	0.05/0.28
FSS	Fosses	France	24	218.30 ± 42.49	25.07/665.76	47.46 ± 3.69	15/79	0.09 ± 0.02	0.01/0.27
GGT	Girgentana	Italy (Sicily)	24	363.58 ± 37.61	161.21/863.58	93.71 ± 2.60	73/122	0.15 ± 0.02	0.07/0.35
ICL	Icelandic goats	Iceland	11	1625.59 ± 57.30	1263.82/1979.66	366.45 ± 12.60	324/455	0.66 ± 0.02	0.51/0.81
MAL	Mallorquina	Spain (Mallorca)	18	334.68 ± 63.06	49.57/938.51	74.39 ± 4.61	29/102	0.14 ± 0.03	0.02/0.38
MEN	Menabe	Madagascar	19	797.36 ± 21.95	673.26/1156.91	392.63 ± 4.70	345/425	0.32 ± 0.01	0.27/0.47
MLS	Maltese Sarda	Italy (Sardinia)	12	287.56 ± 36.34	24.37/486.45	73.42 ± 6.71	18/102	0.12 ± 0.01	0.01/0.20
MOR	Moroccan goat	Morocco	30	151.03 ± 40.45	15.76/1025.09	32.43 ± 2.94	13/74	0.06 ± 0.02	0.01/0.42
MTB	Matebele	Zimbabwe	22	146.35 ± 14.97	110.73/451.20	84.55 ± 1.37	71/96	0.06 ± 0.01	0.05/0.18
MUL	Mulranny	Ireland	13	323.02 ± 33.76	59.55/604.71	74.50 ± 5.42	36/111	0.13 ± 0.01	0.02/0.25
PAL	Palmera	Spain (La Palma)	15	571.80 ± 14.11	471.68/641.05	276.13 ± 4.86	234/314	0.23 ± 0.01	0.19/0.26
RAS	Blanca de Rasquera	Spain	20	246.55 ± 37.98	20.01/588.65	56.45 ± 5.33	17/103	0.10 ± 0.02	0.01/0.24
SAR	Sarda	Italy (Sardinia)	27	97.19 ± 17.14	21.52/376.47	34.81 ± 2.35	14/55	0.04 ± 0.01	0.01/0.15
SOF	Sofia	Madagascar	22	854.77 ± 30.68	672.20/1251.19	363.95 ± 4.65	310/391	0.35 ± 0.01	0.27/0.51
SOU	SudOuest	Madagascar	8	564.73 ± 16.40	488.34/637.01	300.63 ± 7.40	263/325	0.23 ± 0.01	0.20/0.26

### Data analysis

The Zanardi software [22] was used to identify ROH. Runs of homozygosity were defined as homozygous genomic stretches that are at least 1 Mb long and that contain a minimum number of 15 SNPs. We allowed one heterozygous SNP per ROH to account for genotyping errors. Coordinates of principal component analysis (PCA) and allele-sharing distances (ASD) were calculated with the PLINK program [21]. A neighbor-joining tree was built and visualized with the Splitstree program [23].

Genomic inbreeding derived from ROH coverage (*F*_ROH_) was calculated by dividing total ROH length per individual by total genome length across all 29 autosomes (2456.50 Mb). Inbreeding coefficients i.e. *F*_het_, *F*_hat1_, *F*_hat2,_ and *F*_hat3_ were calculated with the PLINK software [21] for populations with at least 20 individuals. On the one hand, the—het command of PLINK [21] was used to compute observed and expected autosomal homozygous genotype counts (*F*_het_) and on the other hand, we used the—ibc command of PLINK [21] to calculate *F*_hat_, *F*_hat2_ and *F*_hat3_ parameters. Observed heterozygosity (H_o_) and effective size (N_e_) parameters were retrieved from estimates provided by Colli and coworkers [16].

Statistical analyses were performed by using the R software v.2.15.3 [24]. The values and statistical significances of Spearman’s rank correlations (ρ) of *F*_ROH_ with H_o_ and N_e_ were computed. We also calculated Spearman’s rank correlations (ρ) of number and total length of ROH for insular goat populations with the distance between each island and the nearest continental location.

We used a generalized least squares model implemented in the *nlme* package (R software v.2.15.3, [24]) to infer whether *F*_ROH_ and total ROH length and number differed significantly between continental and insular populations. We considered six groups of breeds and performed statistical comparisons between the breeds contained within each group (all breeds against each other within each group). We did not perform statistical comparisons between groups or between breeds belonging to different groups. The following groups of breeds were taken into consideration: (1) Icelandic goats, Danish, Dutch and Finnish Landrace; (2) Arran, Bilberry, Mulranny, and Fosses; (3) Argentata, Aspromontana, Ciociara Grigia, Corse, Girgentana, Maltese Sarda and Sarda; (4) Mallorquina and Blanca de Rasquera; (5) Moroccan goat and Palmera; and (6) Androy, Diana, Menabe, Matebele, Sofia and SudOuest. On the basis of exploratory analyses of the data (results not shown), we used a model which assumes inequality of the variances (heteroscedasticity) that are associated with each of the three parameters taken into consideration (*F*_ROH_ and total ROH length and number) and each population:

$${\mathbf{Y}}_{i} = {\mathbf{X}}_{i} {\varvec{\upbeta}} + {\varvec{\upvarepsilon}}_{i} ,\quad {\varvec{\upvarepsilon}}_{i } \sim N \left( {0, \sigma^{2 } {\varvec{\Lambda}}_{i} } \right)\;{\text{and}}\quad i = 1, \ldots ,m,$$

where ${\varvec{\upbeta}}$ is a vector of the fixed effect “breed” (m levels), ${\mathbf{X}}_{i}$ is an incidence matrix relating ${\mathbf{Y}}_{i}$ to ${\varvec{\upbeta}}$, and ${\varvec{\Lambda}}_{i}$ is a positive-definite matrix of the variances and covariances of the within-group errors. For full details on the methodology, see Pinheiro and Bates [25]. The least square means of each parameter and population were contrasted on a pairwise basis with a Wald univariate test of significance [26] and multiple testing was adjusted with the Bonferroni correction.

## Results

**Fig. 1:**
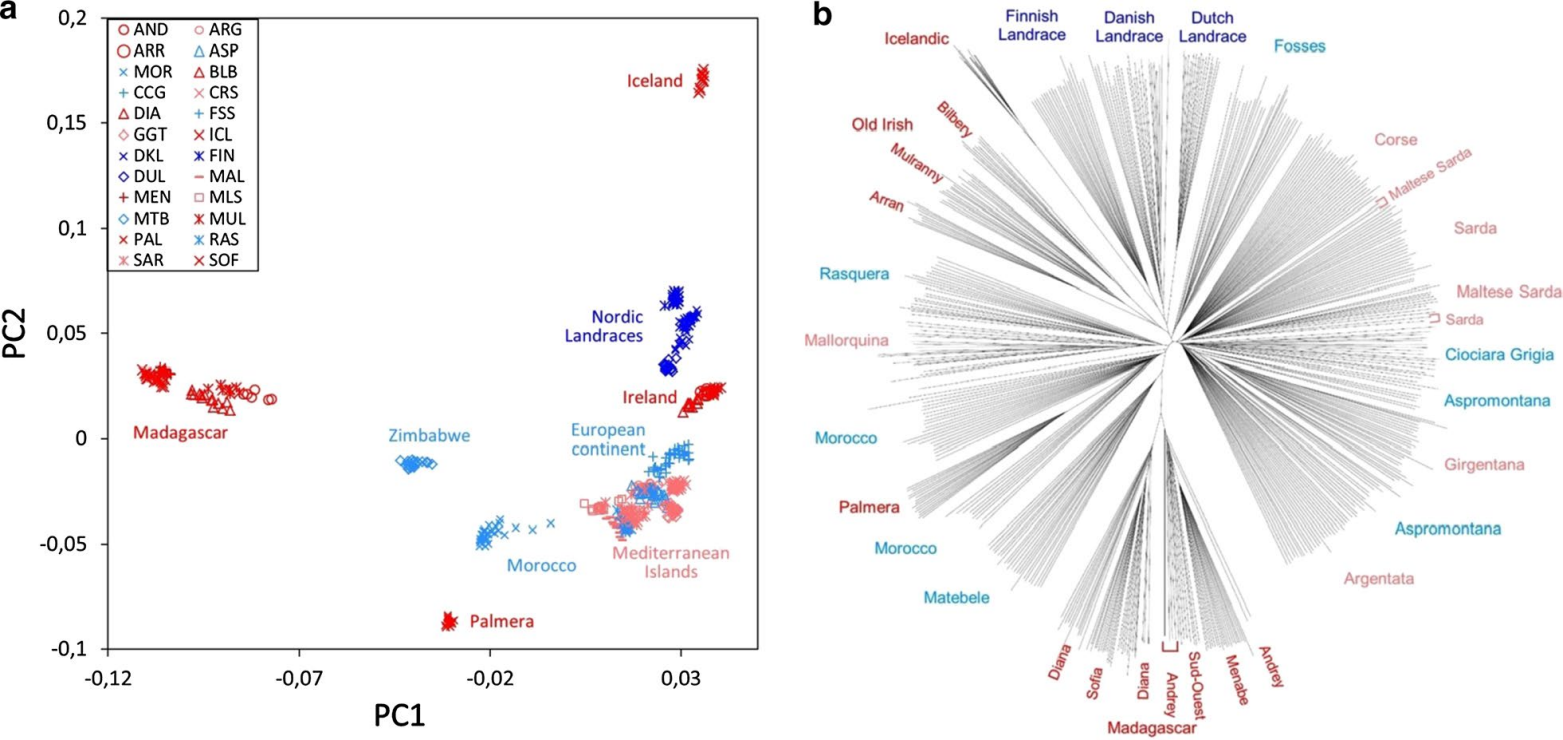
**a** PCA plot of individuals from 25 insular or continental populations. Red and dark blue indicate insular and continental breeds, respectively, with a high level of homozygosity. Pink and light blue indicate insular and continental breeds, respectively, with a low or moderate level of homozygosity. **b** Neighbor-joining tree based on allele-sharing distances representing the genetic relationships among insular and continental goat populations

Our panel of goat breeds included insular breeds from different regions (see Additional file 1: Figure S1): Iceland (N = 11) and Ireland (N = 31) in northern Europe; Corsica (N = 29), Sardinia (N = 39), Sicily (N = 48) and Mallorca (N = 18) in Southern Europe; and La Palma (N = 15) and Madagascar (N = 69) in Africa. Each of these was compared to the nearest continental populations. Among the continental populations, the Finnish, Danish and Dutch goats belong to the Nordic type and are also subject to genetic isolation either because they are bred on a peninsula (Finland, Denmark) or managed under the scope of a strictly closed herdbook (Netherlands). The PCA first factorial map is shown in Fig. 1a. The first two coordinates PC1 and PC2 highlight the separation between Malagasy and Icelandic goats, respectively. These two breeds and the goat populations from La Palma and Iceland, also have extreme positions on the PC1-PC3 and PC1-PC4 plots (see Additional file 2: Figure S2), but this was not observed for goats from Southern European islands (Mallorca, Corsica, Sardinia and Sicily).

A neighbor-joining tree of ASD distances (Fig. 1b) shows a clear clustering of goats from the same breed, except for two Maltese Sarda goats that cluster with the Sarda and vice versa. Girgentana, Palmera and Sofia are nested within the Aspromontana, Morocco and Diana populations, respectively, while both Menabe and Sud-Ouest populations are within the Androy population. Most breeds appear to be homogeneous, except for Danish Landrace, Mallorquina and Aspromontana, which show some heterogeneity. For Icelandic, Irish, Palmera and Malagasy goats, we observed a decrease in genetic distances between individuals from the same breed (Fig. 1b) but not for goats from Mediterranean islands. Apart from the extremely inbred Icelandic goats [12], genetic isolation was most intense for the Irish Arran, Dutch, Palmeran (Canary Islands) and Malagasy (Androy, Sofia, Diana, Menabe and Sud-Ouest) populations. As shown in Figure S3 (see Additional file 3: Figure S3), the within-breed ASD distances correlate closely with H_o_, which indicates that a tree as that shown in Fig. 1b faithfully illustrates the diversity patterns and at the same time reproduces the regional clustering of breeds [16].

**Fig. 2:**
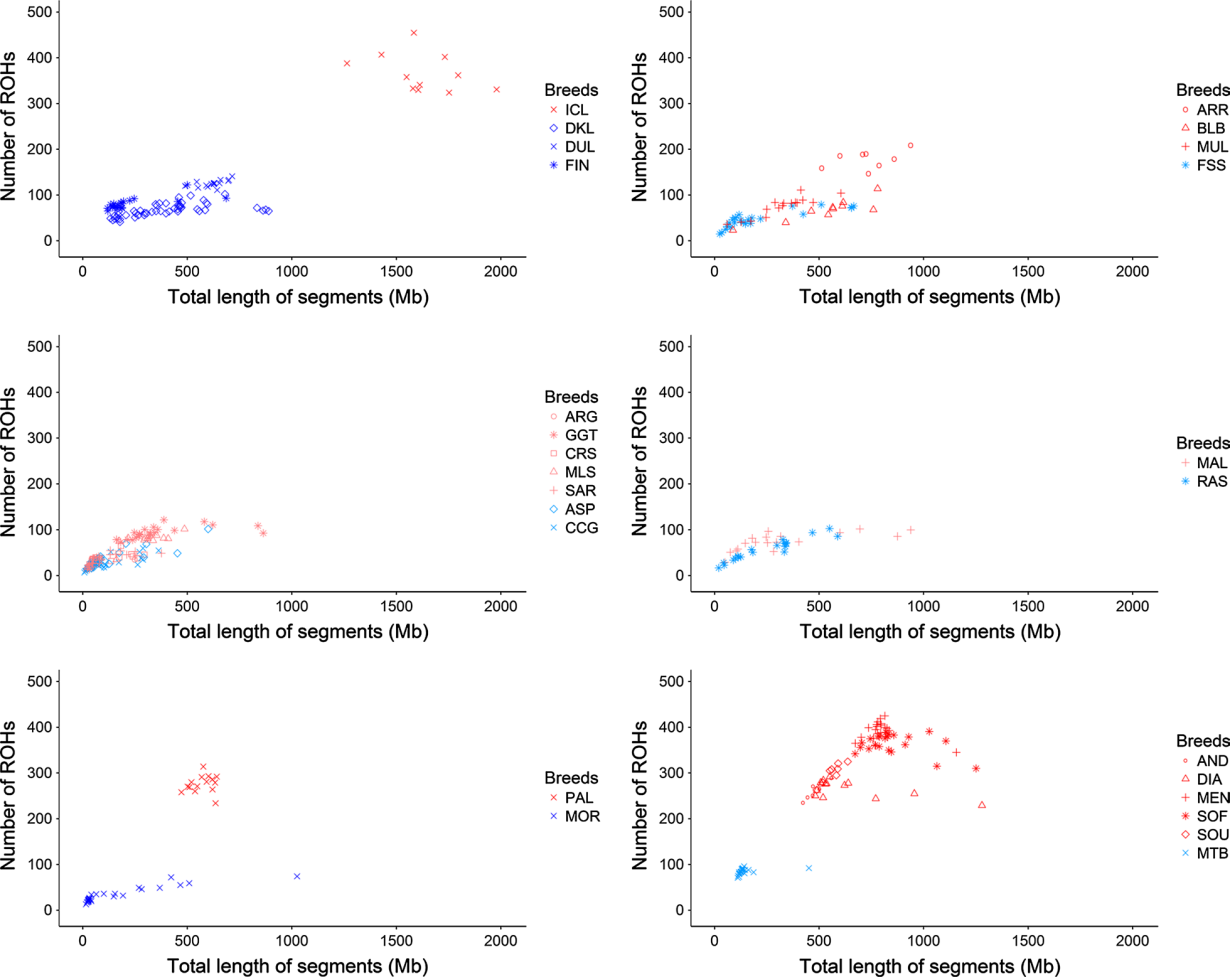
Number and total length of ROH in African, European and Mediterranean insular and continental goat populations. Red and dark blue indicate insular and continental breeds, respectively, with a high level of homozygosity. Pink and light blue indicate insular and continental breeds, respectively, with a low or moderate level of homozygosity. The number of ROH found for each individual genome (*y*-*axis*) is plotted against total ROH size (i.e. number of Mb covered by ROH in each genome, *x*-*axis*). The following codes are used (insular populations in bold): AND, **Androy** (Madagascar); ARG, **Argentata** (Sicily); ARR, **Arran** (Ireland); ASP, Aspromontana; BLB, **Bilberry** (Ireland); CCG, Ciociara Grigia; CRS, **Corse** (Corsica); DIA, **Diana** (Madagascar); DKL, Danish Landrace; DUL, Dutch Landrace; FIN, Finnish Landrace; FSS, Fosses; GGT, **Girgentana** (Sicily); ICL, **Icelandic goats** (Iceland); MAL, **Mallorquina** (Mallorca); MEN, **Menabe** (Madagascar); MLS, **Maltese Sarda** (Sardinia); MOR, Moroccan goat; MTB, Matebele; MUL, **Mulranny** (Ireland); PAL, **Palmera** (La Palma); RAS, Blanca de Rasquera; SAR, **Sarda** (Sardinia); SOF, **Sofia** (Madagascar); SOU, **SudOuest** (Madagascar)

**Fig. 3:**
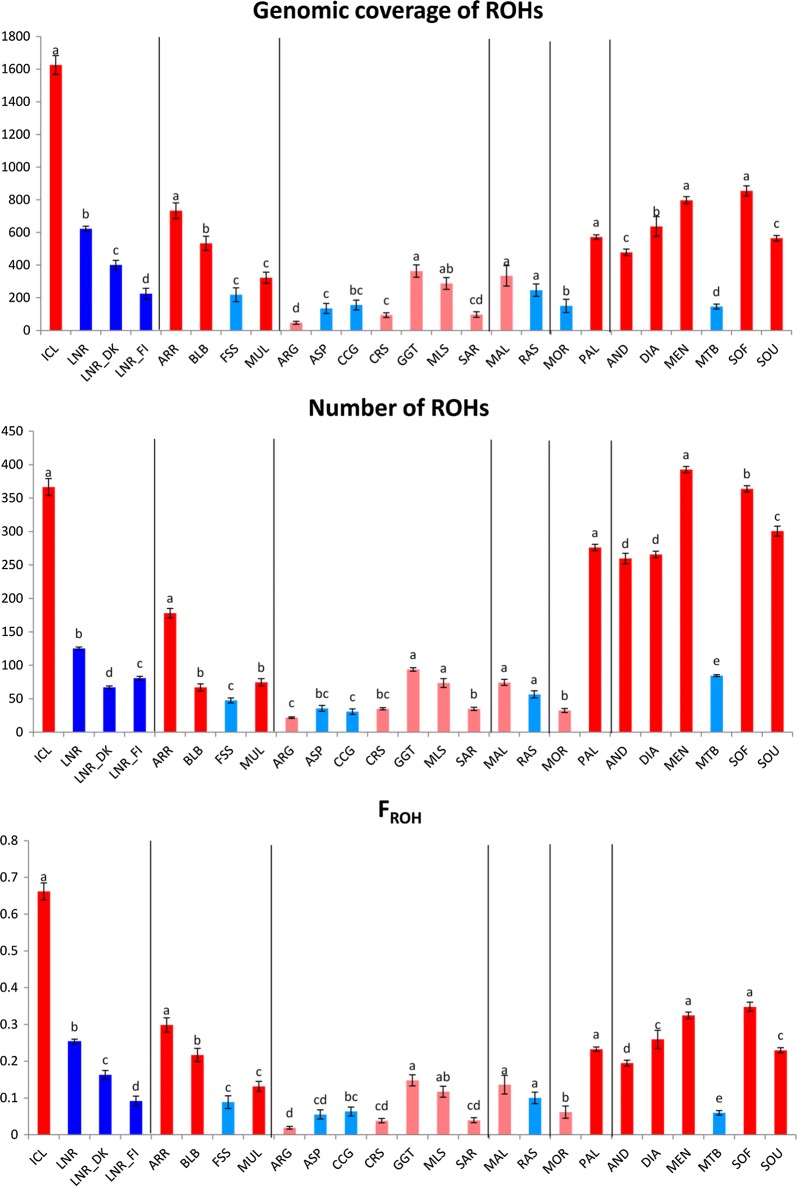
Statistical analysis of the mean ROH numbers, ROH coverage and *F*_ROH_. Red and dark blue indicate insular and continental breeds, respectively, with high homozygosity. Pink and light blue indicate insular and continental breeds, respectively, with low or modest homozygosity. Different letters indicate statistically significant differences between groups (mean ± standard error, linear models with heteroscedastic within group-errors, P_adj_-value < 0.05). The following codes are used (insular populations in bold): AND, **Androy** (Madagascar); ARG, **Argentata** (Sicily); ARR, **Arran** (Ireland); ASP, Aspromontana; BLB, **Bilberry** (Ireland); CCG, Ciociara Grigia; CRS,** Corse** (Corsica); DIA, **Diana** (Madagascar); DKL, Danish Landrace; DUL, Dutch Landrace; FIN, Finnish Landrace; FSS, Fosses; GGT, **Girgentana** (Sicily); ICL,** Icelandic goats** (Iceland); MAL, **Mallorquina** (Mallorca); MEN, **Menabe** (Madagascar); MLS, **Maltese Sarda** (Sardinia); MOR, Moroccan goat; MTB, Matebele; MUL, **Mulranny** (Ireland); PAL, **Palmera** (La Palma); RAS, Blanca de Rasquera; SAR, **Sarda** (Sardinia); SOF, **Sofia** (Madagascar); SOU, **SudOuest** (Madagascar)

The number of ROH and the total ROH coverage are displayed in Fig. 2 and the means and standard errors of these two parameters are in Fig. 3 and Table 1. Significant differences in ROH coverage, ROH number and *F*_ROH_ (P_adj_-value < 0.05) were observed between goats from Iceland, Madagascar, La Palma and Ireland (Bilberry and Arran) and their continental counterparts (Fig. 3). Goats from Mediterranean islands represent a more complex case because certain populations display a significantly increased level of homozygosity (e.g. Girgentana) while others do not (e.g. Corse and Argentata).

**Fig. 4:**
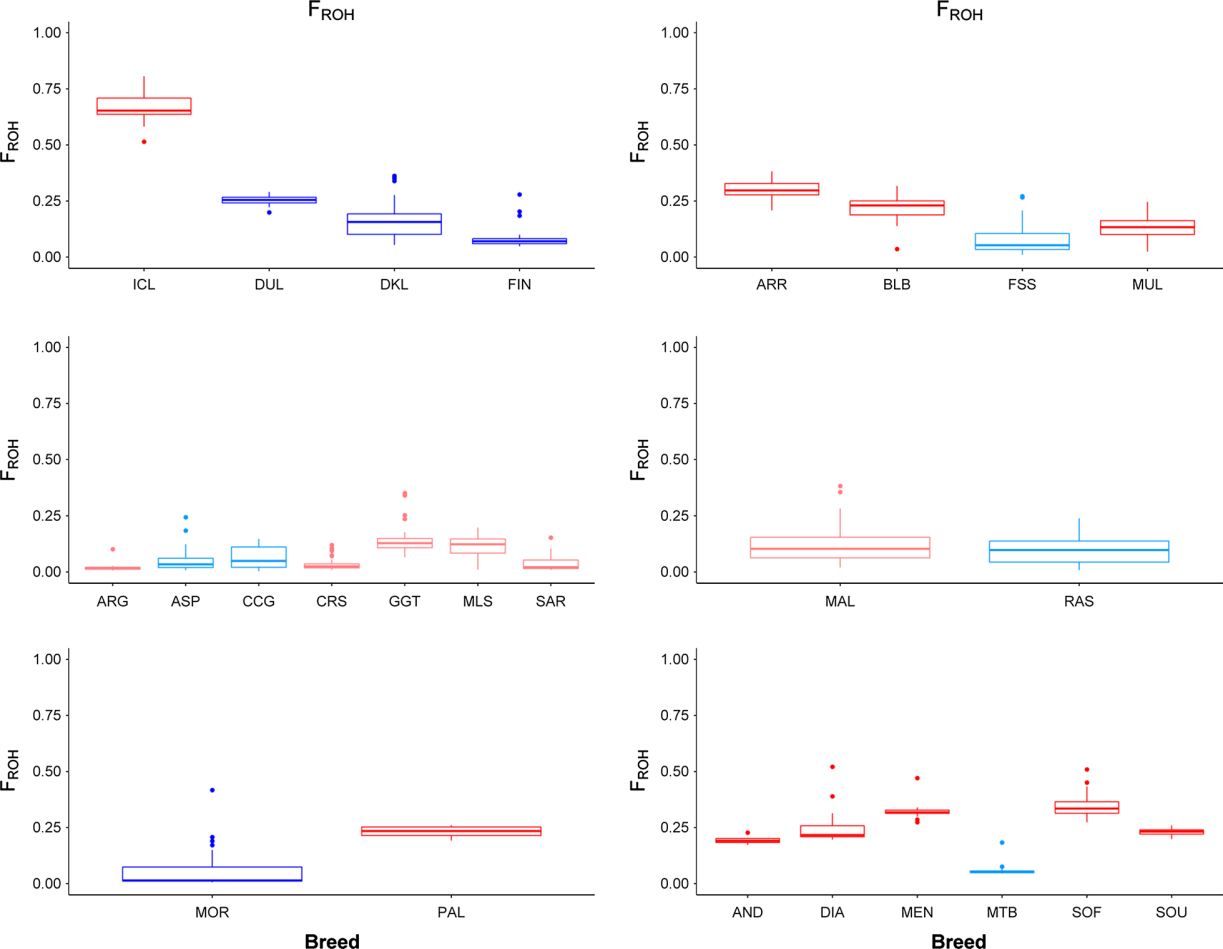
Plot of the fraction of the genome covered by ROH (*F*_ROH_) in insular (red) and continental (blue) goat populations. The following codes are used (insular populations in bold): AND, **Androy** (Madagascar); ARG, **Argentata** (Sicily); ARR, **Arran** (Ireland); ASP, Aspromontana; BLB, **Bilberry** (Ireland); CCG, Ciociara Grigia; CRS, **Corse** (Corsica); DIA, **Diana** (Madagascar); DKL, Danish Landrace; DUL, Dutch Landrace; FIN, Finnish Landrace; FSS, Fosses; GGT, **Girgentana** (Sicily); ICL, **Icelandic goats** (Iceland); MAL, **Mallorquina** (Mallorca); MEN, **Menabe** (Madagascar); MLS, **Maltese Sarda** (Sardinia); MOR, Moroccan goat; MTB, Matebele; MUL, **Mulranny** (Ireland); PAL, **Palmera** (La Palma); RAS, Blanca de Rasquera; SAR, **Sarda** (Sardinia); SOF, **Sofia** (Madagascar); SOU, **SudOuest** (Madagascar)

Genomic ROH coverage is directly proportional to the ROH-based inbreeding coefficients [27] that are shown in Fig. 4 and Table 1. In the Icelandic, Arran (Ireland), Malagasy and Palmeran goat populations, the number of ROH and their total length range from 147 to 455 and from 423.28 to 1979.66 Mb (Fig. 4 and Table 1), respectively, which correspond to extremely high inbreeding coefficients in Icelandic (*F*_ROH_ = 0.66), and Malagasy goats (*F*_ROH_ = 0.19–0.35). The genetic isolation of the Dutch goats [14] is evidenced by a high ROH coverage ranging from 133.11 to 889.28 Mb (*F*_ROH_ = 0.25) rather than by the number of ROH (41–102). Inbreeding coefficients are also high for the Palmeran (*F*_ROH_ = 0.23), Bilberry (*F*_ROH_ = 0.22) and Arran (*F*_ROH_ = 0.30) populations (Fig. 4 and Table 1). In contrast, Mediterranean insular goats have at most 122 ROH with a mean total length of 47.1–363.6 Mb (Fig. 2 and Table 1), these values being similar to those observed for nearby continental goat populations (Fig. 3). A total length of ROH greater than 500 Mb was observed only for a few Girgentana and Mallorquina goats.

**Fig. 5:**
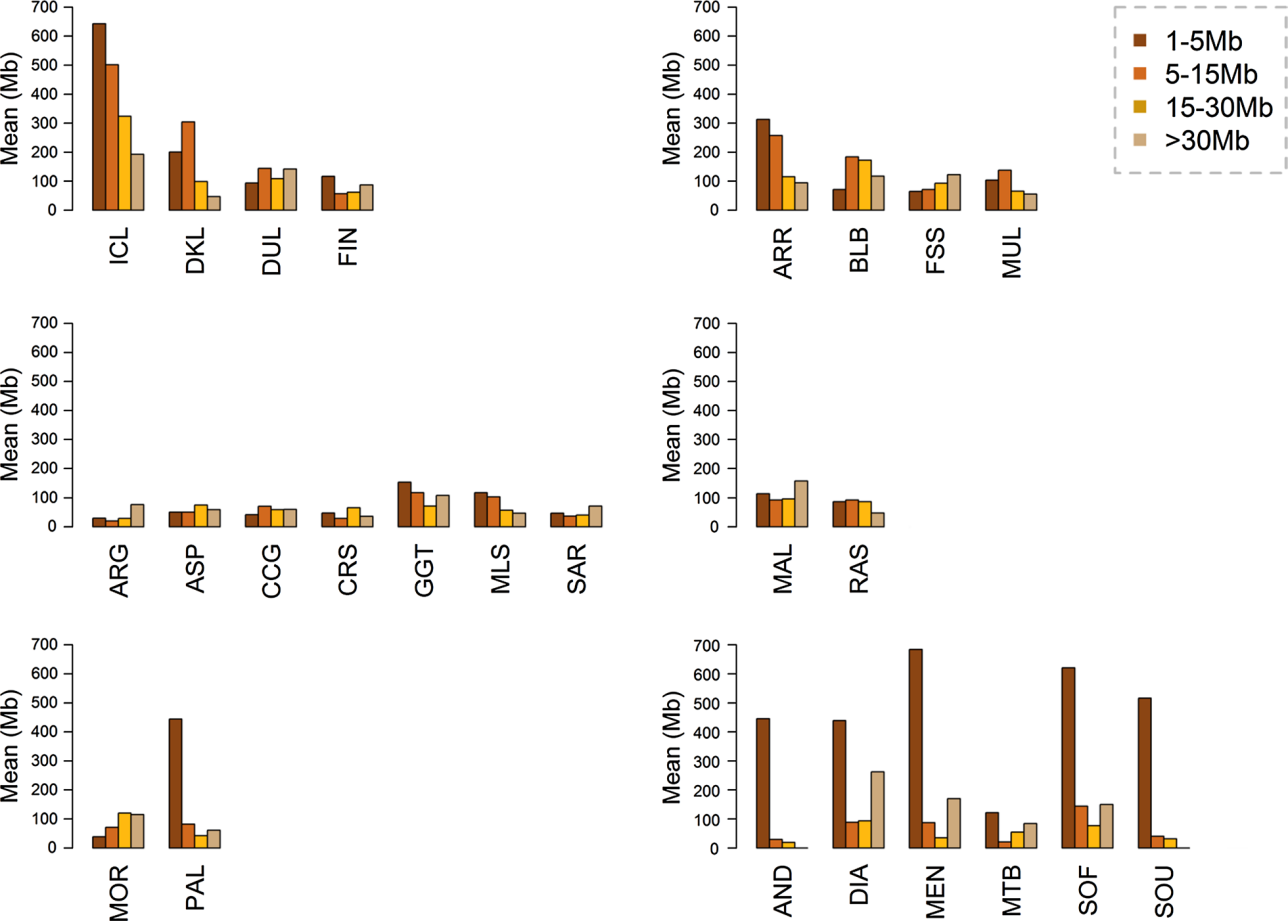
Distribution of ROH according to their size in African, European and Mediterranean insular and continental goat populations. The following codes are used (insular populations in bold): AND, **Androy** (Madagascar); ARG, **Argentata**; ARR, **Arran** (Ireland); ASP, Aspromontana; BLB, **Bilberry** (Ireland); CCG, Ciociara Grigia; CRS, **Corse** (Corsica); DIA, **Diana** (Madagascar); DKL, Danish Landrace; DUL, Dutch Landrace; FIN, Finnish Landrace; FSS, Fosses; GGT, **Girgentana** (Sicily); ICL, **Icelandic goats** (Iceland); MAL, **Mallorquina** (Mallorca); MEN, **Menabe** (Madagascar); MLS, **Maltese Sarda** (Sardinia); MOR, Moroccan goat; MTB, Matebele; MUL, **Mulranny** (Ireland); PAL, **Palmera** (La Palma); RAS, Blanca de Rasquera; SAR, **Sarda** (Sardinia); SOF, **Sofia** (Madagascar); SOU, **SudOuest** (Madagascar)

In most breeds, we found an inverse relationship between ROH size and frequency (Fig. 5). However, ROH longer than 1–5 Mb are relatively frequent in the genome of Icelandic, Irish, Dutch, Danish and Mallorquina goats. For the extremely inbred Iceland goat, an average of 366 ROH were detected, 286 of which were longer than 1 to 5 Mb. We also investigated the relationship between *F*_ROH_ and several population parameters including (H_o_, N_e_ and the inbreeding coefficients *F*_het_, *F*_hat1_, *F*_hat2_ and *F*_hat3_ calculated with PLINK [21] i.e. (see Additional file 4: Table S1 and Additional file 5: Table S2). On the one hand, Spearman correlations coefficients between *F*_ROH_ and N_e_ and H_o_ were negative (ρ < − 0.87) and significant (*P* value < 0.002), which reflects that populations with smaller N_e_ undergo more genetic drift than larger populations [27] and, in consequence, they tend to be more homozygous. On the other hand, Spearman correlations coefficients between *F*_ROH_ and the four inbreeding coefficients calculated with PLINK [21] were positive (ρ < 0.64–0.88) and significant (*P* value < 0.01) or very significant (*P* value < 0.0001). Previous studies have reported similar trends for correlations between *F*_ROH_ and a wide range of inbreeding coefficients [27–29].

We also calculated Spearman correlations coefficients of ROH length and number with the distance of insular goat populations to the nearest continental coastal site. For goats raised in distant islands, such as Iceland (1277 km from Norway) or Madagascar (1051 km from Mozambique), ROH number and total length had the largest values. Spearman correlation coefficients of the distances with ROH number (ρ = 0.54 and, *P* value = 0.023) and total length (ρ = 0.63 and, *P* value = 0.007) were positive and significant.

## Discussion

In insular and other isolated populations, homozygosity is often increased by founder effects and geographic isolation. In our study, the larger numbers and longer total lengths of ROH and higher *F*_ROH_ in goats from Iceland, Ireland, La Palma and Madagascar than in their continental counterparts illustrate this increase in the level of homozygosity (Table 1 and Figs. 2, 3 and 4). In contrast, Mediterranean insular and continental populations form a tight cluster. Although goat populations that are raised in remote islands tend to have higher levels of homozygosity, other factors are also involved, such as breed management, history and demography, which could have strong effects on breed diversity. We were not able to analyse the impact of some of these factors because of lack of information (historic and demographic records are scarce or completely absent for most of the breeds under analysis).

The analysis illustrated in Fig. 1a reveals a strong separation between Malagasy and Matebele (Zimbabwe) and between Icelandic and Nordic Landrace goat populations and, to a lesser extent, between Palmeran and Moroccan goats. This result probably reflects the extreme geographic isolation of these three insular populations. Indeed, we found that ROH number and total length were correlated positively with distance between each island and the nearest continental location. Effects of insularization and/or small effective populations sizes were previously reported in a comparison between Japanese wild boars and Asian mainland pigs [2], and for several insular and/or inbred cattle populations [28].

We observed the most extreme combined effects of founder events, geographic isolation and small population size for the Icelandic goats with 324 to 455 ROH that cover between 1263 and 1979 Mb, a high frequency of very long ROH (> 30 Mb) and a very high *F*_ROH_ of 0.66 (Table 1 and Figs. 3, 4, 5). Icelandic goats have a North-European (most likely Norwegian) origin and were imported during the colonization of Iceland over 1100 years ago [12, 29]. The lack of evidence for subsequent goat importations suggests a scenario of strong geographic isolation. Moreover, there were less than 100 Icelandic goats at the end of the nineteenth century and again in 1960, but, in 1994, the estimated census was equal to 348 heads distributed over 48 flocks [29]. Small population size combined with a fragmented distribution probably favoured the maintenance of high levels of inbreeding in this goat population. Old Irish goats, most notably those from Arran and Bilberry, also show increased ROH coverage, but not as extreme as that observed in Icelandic goats (Figs. 3, 4, 5). The extremely small population (27 in 2006, [30]) of Bilberry goats has led to the emergence of relatively long ROH (Figs. 3, 5), which indicates recent consanguinity. Old Irish goats have been subjected to casual hunting and indiscriminate culling of feral herds, which has led this population to the verge of extinction [31].

Increased homozygosity was also observed in goat populations that have large population sizes but, as for the Icelandic and Irish goats, have endured a prolonged geographic isolation. For both Palmera and Malagasy goats, a relatively large number of ROH, high ROH coverage (Fig. 2) and elevated *F*_ROH_ (0.19–0.35, Table 1) were found, but short ROH (1–5 Mb) were predominant (Fig. 5). This is probably the consequence of an ancient founder effect and geographic isolation, whereas their large population size (1.2 million in Madagascar and more than 6000 in La Palma) prevents consanguinity and the generation of long ROH. Madagascar, the fourth largest island in the world, was settled by Austronesians, who arrived from Borneo during the fifth to seventh centuries, and subsequently by Bantu people [32], but it is clear that Malagasy goats are of African origin (Fig. 1). The patterns of ROH observed in our study suggest that, after an initial founder effect, Malagasy goats were subject to a history of prolonged geographic isolation, with the distance of 1000 km between Madagascar and the African landmass constituting an effective barrier to gene flow. La Palma, in the Canarian archipelago, was settled 2500 YBP by colonists with probably a Berber ancestry and remained isolated from the main maritime routes until its colonization by the Spanish in the fifteenth century [33]. This North African ancestry of Palmeran goats is reflected in the neighbor-joining tree shown in Fig. 1, with Palmeran goats displaying a close relationship with Moroccan goats. In the La Palma island, a limited number of founders in combination with geographic isolation resulted in a low level of diversity of caprine mtDNA [34] and a high ROH coverage.

The above results are in strong contrast with the majority of the Mediterranean insular goat breeds showing ROH patterns and *F*_ROH_ values that are similar to those observed in nearby continental populations (Table 1 and Figs. 2, 3, 4, 5). Mallorca, Corsica, Sicily and Sardinia are relatively close (5–300 km) to continental Europe and are located along maritime routes that were used intensively by the Phoenicians, Carthaginians, Greeks, Romans, Arabs and many other seafaring civilizations [35]. This situation probably favoured the recurrent admixture of goat populations with different genetic backgrounds, thus counteracting the decrease in genetic variation produced by the initial founder effect. This is also illustrated by the segregation of the mtDNA G haplogroup in Mallorquina goats, which so far has only been identified in goats from Egypt, Iran and Turkey [34]. Furthermore, a mixed ancestry with a major influence of Maltese goats has been mentioned for Sarda goat [36]. However, Mallorquina and Girgentana goats are exceptions with a high frequency of long ROH (> 30 Mb, Fig. 5) and *F*_ROH_ values of 0.14 and 0.15, respectively. The highly endangered Mallorquina goats have suffered strong population bottlenecks (current census = 150 individuals) [34]. The Girgentana breed has also experienced a strong demographic recession from 30,000 individuals in 1983 to 461 in 1993 [37]. Thus, for both these breeds, long ROH (> 30 Mb) are explained by population bottlenecks and recent inbreeding.

As illustrated by the Finnish, Danish and Dutch Landrace breeds, genetic isolation may also occur in continental populations that are raised in remote locations from Finland and Denmark or subject to a strict breeding management and selection in combination with the occurrence of founder effects (Netherlands). The Dutch goat population has been revived since 1958: it started with two remaining individuals, involved undocumented crossbreeding and resulted in the current population of ca. 2000 animals [14]. Genetic distances show a relatedness to the Danish and Finnish Landrace populations, which was not detected by a panel of 26 microsatellites [14]. Remarkably, the numbers and length distributions of ROH for these populations are similar to those reported for British cattle breeds Angus, Hereford, Jersey and Guernsey [28], and larger than those reported for central European continental goats (this study) or European continental cattle [28].

A common finding in several studies of ROH [14, 28, 38] is the considerable variation in patterns of ROH within breeds. We observed this also in our dataset, in spite of the high level of within-breed homogeneity (Fig. 1b). The genomic coverage of ROH—up to 1.6 Gb in the Iceland breed and less than 100 Mb only for three breeds—indicates that a substantial part of the genes are homozygous, which may have detrimental consequences on the biological viability of isolated populations due to inbreeding depression and increased frequency of recessive hereditary diseases [1, 9, 11]. However, small population sizes promote the loss of recessive gene defects. Thus, future work should investigate the consequences of high ROH coverage based on whole-genome sequence data.

## Conclusions

Our data show that insularization generally involves increased levels of homozygosity. At the same time, patterns of ROH are highly divergent among insular (and also continental) goat breeds: whereas goats that are raised in Madagascar, Iceland and La Palma, show high levels of homozygosity, those bred in Mediterranean islands display homozygosity patterns that are comparable to those found in continental populations. These results indicate that the effects of isolation are modulated by a complex network of factors including population size, breed history and demography, geographic distribution, trading and breed management, which either maintain strict isolation or allow cross-breeding.

## Additional files

**Additional file 1: Figure S1.** Geographic locations of the insular and continental breeds considered in our study. Red and dark blue indicate insular and continental breeds, respectively, with high homozygosity. Pink and light blue indicate insular and continental breeds, respectively, with low or modest homozygosity.

**Additional file 2: Figure S2.** PCA plot of 25 insular and continental goats for coordinate PC1 against PC2, PC3 and PC4 averaged across individuals. Red and dark blue indicate insular and continental breeds, respectively, with high homozygosity. Pink and light blue indicate insular and continental breeds, respectively, with low or modest homozygosity.

**Additional file 3: Figure S3.** Relationship between observed heterozygosity versus (left) ROH coverage or (right) within-population allele-sharing distances. Red and dark blue indicate insular and continental breeds, respectively, with high homozygosity. Pink and light blue indicate insular and continental breeds, respectively, with low or modest homozygosity.

**Additional file 4: Table S1.** Spearman correlation analysis between genomic inbreeding derived from ROH coverage (F_ROH_) and observed heterozygosity (H_o_), effective size (N_e_) and inbreeding coefficients calculated with PLINK i.e. *F*_het_, *F*_hat1_, *F*_hat2_ and *F*_hat3_. The data represent the statistical analysis between genomic inbreeding and inbreeding coefficients.

**Additional file 5: Table S2.** Inbreeding coefficients (i.e. *F*_het_, *F*_hat1_, *F*_hat2_ and *F*_hat3_) for populations with at least 20 individuals calculated with the PLINK software. The data represent the inbreeding coefficients calculated with PLINK software for different populations analysed in this work.
