# Supplementary material for: Patterns of homozygosity in insular and continental goat breeds
Source: Genet Sel Evol. 2018 Nov 19;50:56. doi: 10.1186/s12711-018-0425-7 (PMC6241035; doi:10.1186/s12711-018-0425-7)
Supplement: Supplementary file 1 — Additional file 1: Figure S1. Geographic locations of the insular and continental breeds considered in our study. Red and dark blue indicate insular and continental breeds, respectively, with high homozygosity. Pink and light blue indicate insular and continental breeds, respectively, with low or modest homozygosity. [file 12711_2018_425_MOESM1_ESM.pptx]

## Slide 1
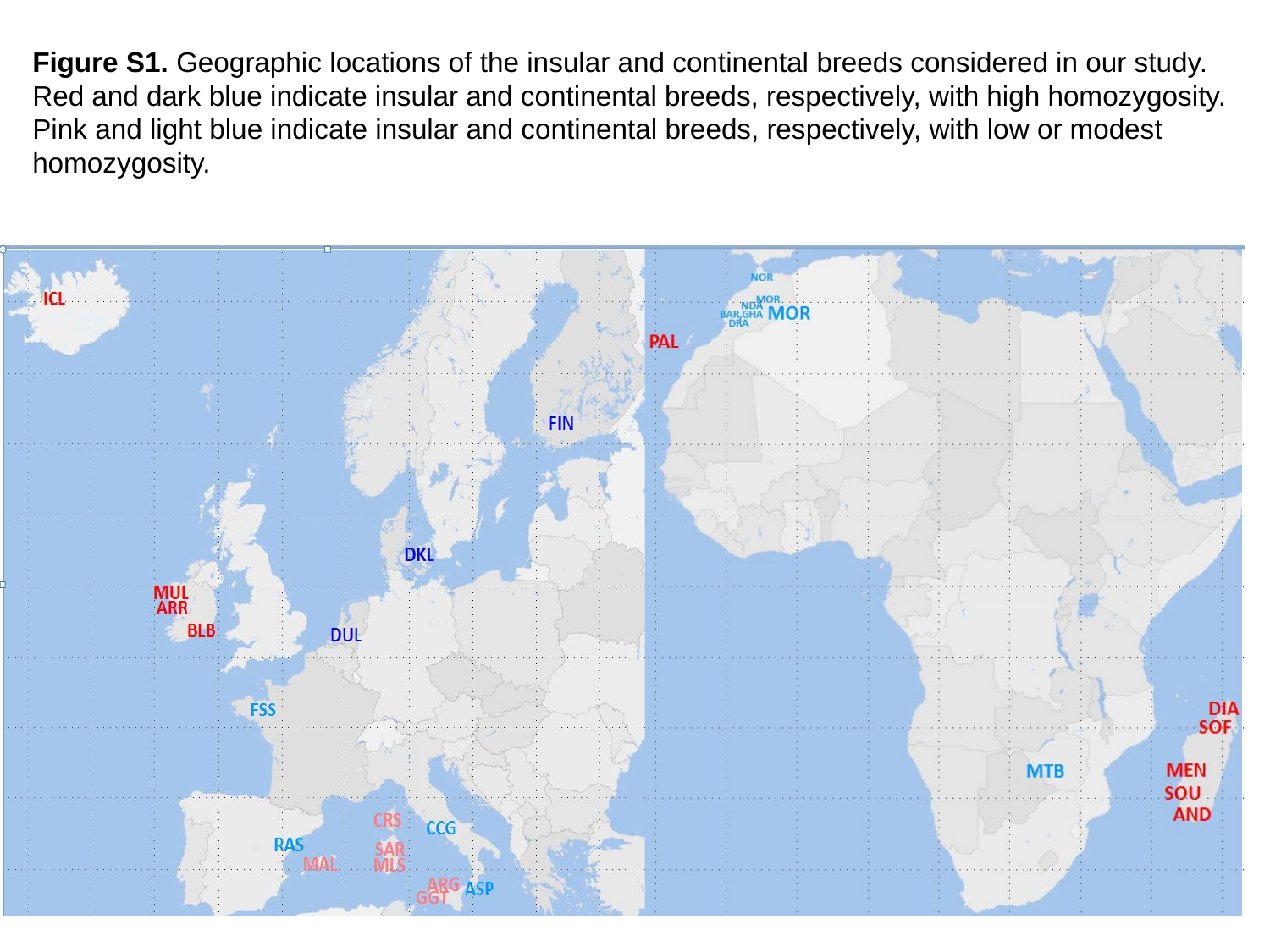

Figure S1. Geographic locations of the insular and continental breeds considered in our study. Red and dark blue indicate insular and continental breeds, respectively, with high homozygosity. Pink and light blue indicate insular and continental breeds, respectively, with low or modest homozygosity.
