# Supplementary material for: Patterns of homozygosity in insular and continental goat breeds
Source: Genet Sel Evol. 2018 Nov 19;50:56. doi: 10.1186/s12711-018-0425-7 (PMC6241035; doi:10.1186/s12711-018-0425-7)
Supplement: Supplementary file 2 — Additional file 2: Figure S2. PCA plot of 25 insular and continental goats for coordinate PC1 against PC2, PC3 and PC4 averaged across individuals. Red and dark blue indicate insular and continental breeds, respectively, with high homozygosity. Pink and light blue indicate insular and continental breeds, respectively, with low or modest homozygosity. [file 12711_2018_425_MOESM2_ESM.pptx]

## Slide 1
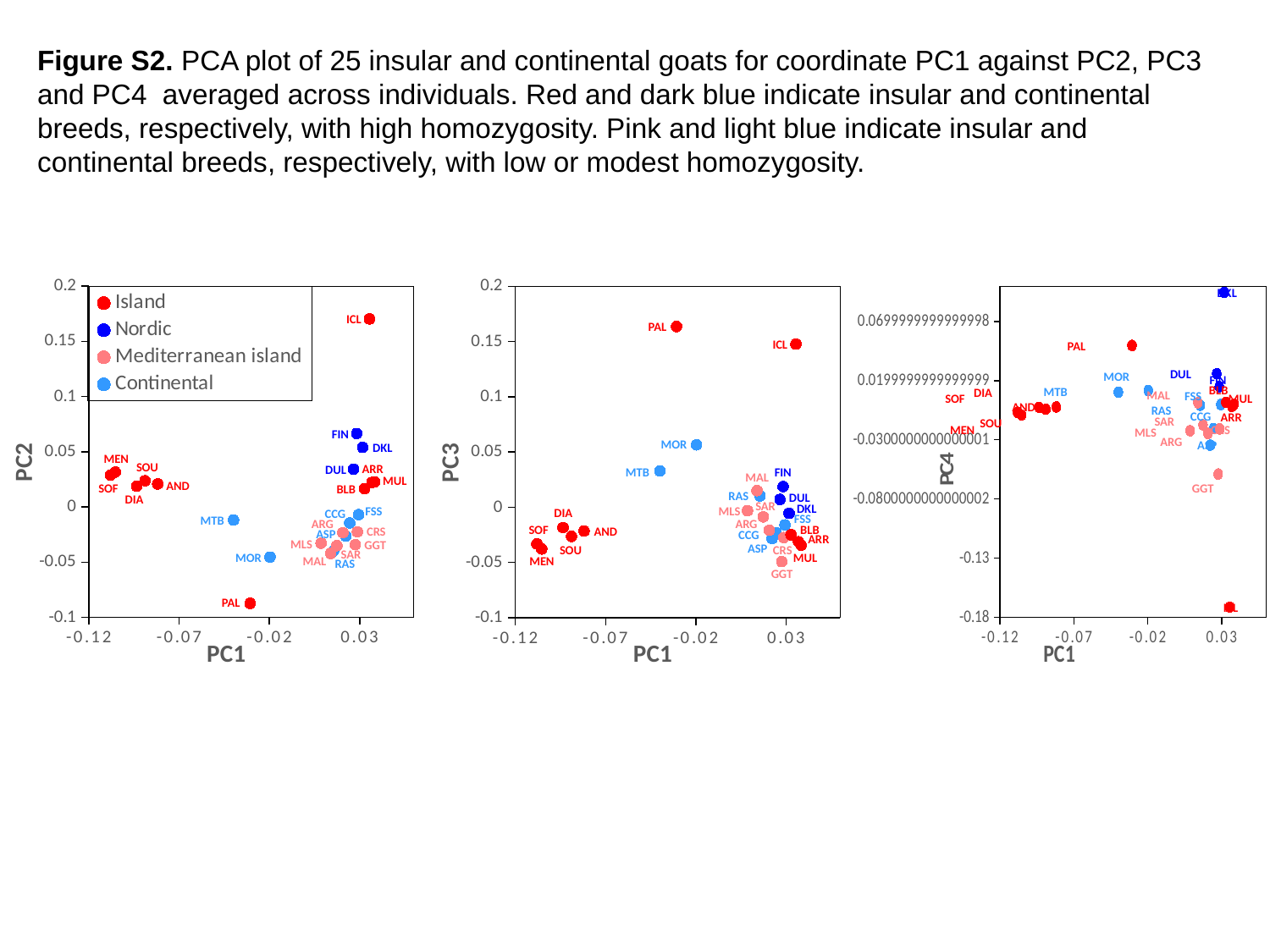

Figure S2. PCA plot of 25 insular and continental goats for coordinate PC1 against PC2, PC3 and PC4 averaged across individuals. Red and dark blue indicate insular and continental breeds, respectively, with high homozygosity. Pink and light blue indicate insular and continental breeds, respectively, with low or modest homozygosity.
### Chart
| Category | Island | Nordic | Mediterranean island | Continental |
|---|---|---|---|---|
### Chart
| Category | Island | Nordic | Mediterranean island | Continental |
|---|---|---|---|---|
### Chart
| Category | Island | Nordic | Mediterranean island | Continental |
|---|---|---|---|---|DKL
ICL
PAL
ICL
PAL
DUL
MOR
FIN
BLB
MTB
DIA
MAL
FSS
SOF
MUL
AND
RAS
CCG
ARR
SAR
SOU
MEN
CRS
MLS
FIN
ARG
MOR
ASP
DKL
MEN
SOU
ARR
DUL
MTB
FIN
MAL
MUL
AND
GGT
SOF
BLB
RAS
DUL
DIA
SAR
DKL
FSS
MLS
DIA
CCG
FSS
MTB
ARG
ARG
BLB
SOF
CRS
AND
ASP
CCG
ARR
MLS
GGT
ASP
SOU
CRS
SAR
MOR
MUL
MAL
MEN
RAS
GGT
PAL
ICL
