# Supplementary material for: Patterns of homozygosity in insular and continental goat breeds
Source: Genet Sel Evol. 2018 Nov 19;50:56. doi: 10.1186/s12711-018-0425-7 (PMC6241035; doi:10.1186/s12711-018-0425-7)
Supplement: Supplementary file 3 — Additional file 3: Figure S3. Relationship between observed heterozygosity versus (left) ROH coverage or (right) within-population allele-sharing distances. Red and dark blue indicate insular and continental breeds, respectively, with high homozygosity. Pink and light blue indicate insular and continental breeds, respectively, with low or modest homozygosity. [file 12711_2018_425_MOESM3_ESM.pptx]

## Slide 1
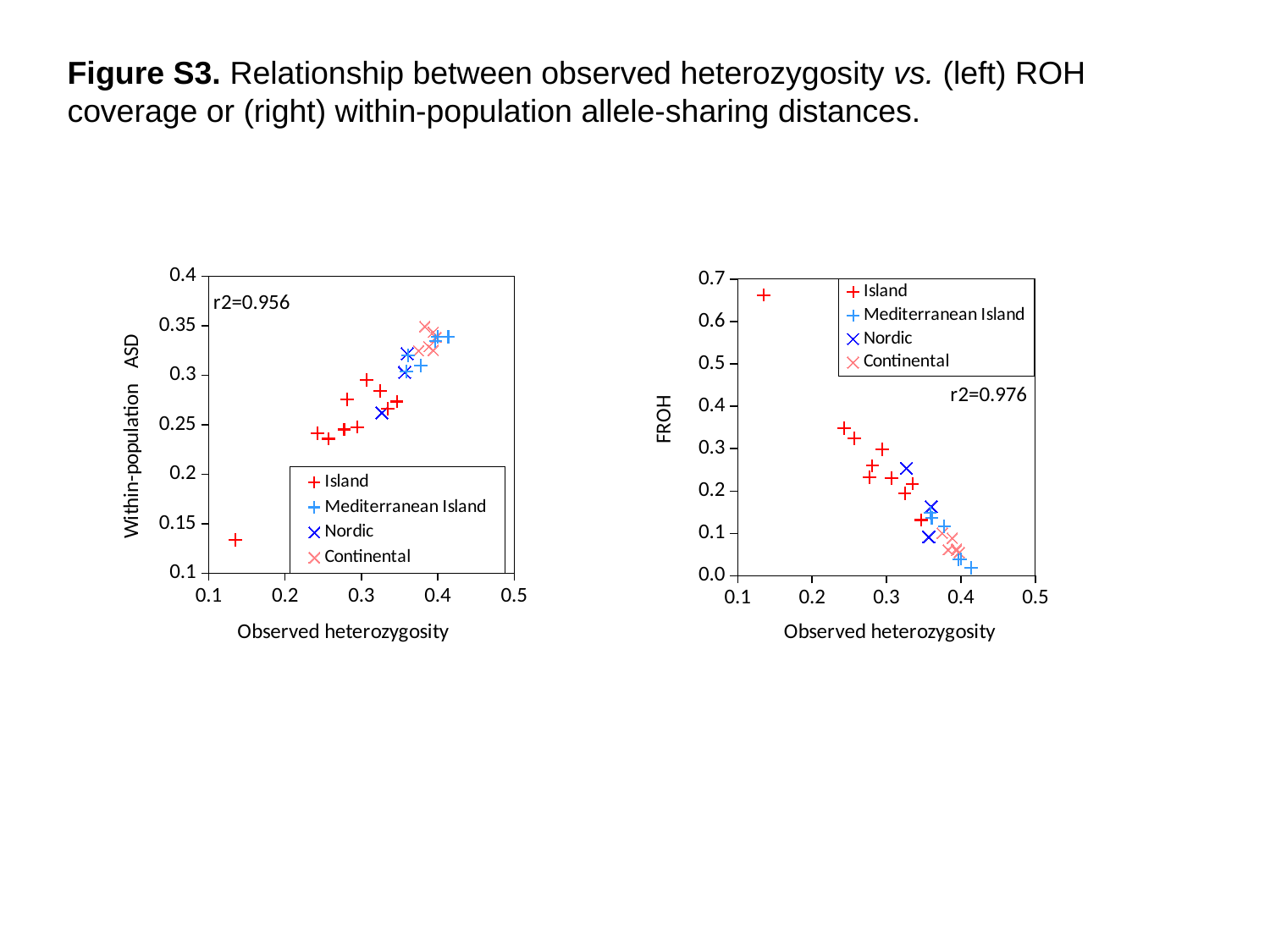

Figure S3. Relationship between observed heterozygosity vs. (left) ROH coverage or (right) within-population allele-sharing distances.
### Chart: r2=0.956
| Category | Island | Mediterranean Island | Nordic | Continental |
|---|---|---|---|---|
### Chart: r2=0.976
| Category | Island | Mediterranean Island | Nordic | Continental |
|---|---|---|---|---|
